# How Are Health Research Priorities Set in Low and Middle Income Countries? A Systematic Review of Published Reports

**DOI:** 10.1371/journal.pone.0108787

**Published:** 2014-10-02

**Authors:** Skye McGregor, Klara J. Henderson, John M. Kaldor

**Affiliations:** The Kirby Institute, The University of New South Wales, Sydney, NSW, Australia; University of Oxford, Kenya

## Abstract

**Background:**

Priority setting is increasingly recognised as essential for directing finite resources to support research that maximizes public health benefits and drives health equity. Priority setting processes have been undertaken in a number of low- and middle-income country (LMIC) settings, using a variety of methods. We undertook a critical review of reports of these processes.

**Methods and Findings:**

We searched electronic databases and online for peer reviewed and non-peer reviewed literature. We found 91 initiatives that met inclusion criteria. The majority took place at the global level (46%). For regional or national initiatives, most focused on Sub Saharan Africa (49%), followed by East Asia and Pacific (20%) and Latin America and the Caribbean (18%). A quarter of initiatives aimed to cover all areas of health research, with a further 20% covering communicable diseases. The most frequently used process was a conference or workshop to determine priorities (24%), followed by the Child Health and Nutrition Initiative (CHNRI) method (18%). The majority were initiated by an international organization or collaboration (46%). Researchers and government were the most frequently represented stakeholders. There was limited evidence of any implementation or follow-up strategies. Challenges in priority setting included engagement with stakeholders, data availability, and capacity constraints.

**Conclusions:**

Health research priority setting (HRPS) has been undertaken in a variety of LMIC settings. While not consistently used, the application of established methods provides a means of identifying health research priorities in a repeatable and transparent manner. In the absence of published information on implementation or evaluation, it is not possible to assess what the impact and effectiveness of health research priority setting may have been.

## Introduction

Health research is an essential tool for improving health and health equity in low- and middle-income countries (LMICs) [1], [2]. Research has the potential to deliver widespread population health changes that respond to critical needs and contribute to sustainable development outcomes in the world's poorest [1]. The 2013 World Health Report highlights the essential role health research plays in progressing the Millennium Development Goals and universal health coverage [2]. In resource rich settings, a high proportion of available research funds go to investigator driven initiatives, but in LMICs there is an expectation that research must respond more directly to community health needs, and therefore be conducted according to recognised priorities.

In the absence of priority setting, there is a risk that research conducted in LMICs will follow topics determined by funders for their own purposes [3] or fail to respond to explicit health needs. Alignment with donor policies can distort national priorities and undermine the role of national research in LMICs [4]. In an essay by Sridhar, developing country health ministers have argued that research priority setting in LMCs is also affected by ‘multi-bi financing’, which is the practice whereby donors choose to route earmarked funding through multilateral agencies and new multistakeholder initiatives (such as the Global Fund to Fight AIDS, Tuberculosis and Malaria). ‘Multi-bi financing’ risks imposing the priorities of powerful states on poorer countries weakening the opportunity for national priority-setting [3].

In 1990 the Commission on Health Research and Development drew attention to the need for Essential National Health Research for LMICs [5]. Over the last three decades methods of health research priority setting (HRPS) have evolved in response, with numerous approaches being taken. The publication in 2000 of the 10/90 Report on Health Research [6], highlighting the gap in expenditure on diseases that affect the world's poorest, provided further impetus to strengthen health research priority setting in LMICs.

There are several different established methodologies that have been employed for health research priority setting [2]. Comprehensive approaches [7] include the 3-Dimensional Combined Approach Matrix [8]–[11] (3D CAM), the Essential National Health Research (ENHR) method [10], [12], the Child Health and Nutrition Research Initiative (CHNRI) method [9], [10], [13] and the Council on Health Research and Development (COHRED) method [10]. Other approaches used include the Delphi method [14] where stakeholders develop an initial list, which is recirculated for further consideration, and the nominal group technique whereby consensus is reached through discussion [15]. Other, more informal methodologies have included a stepwise process, which may consist of an initial literature review, qualitative collection of data through interviews and focus groups, and prioritisation process through a workshop or further consultation with stakeholders; and national or regional workshops/conferences without any explicit specification of a pre-defined HRPS strategy. A number of articles provide detailed analysis of the different methods and approaches [7], [10], [16].

Previous reviews of research priority setting methodology have been restricted to geographic areas [17], [18]; specific areas of health research, such as child and maternal health [19]–[21], tuberculosis [20], mental health [22], and health systems research [23], [24]; national level exercises [25], [26] or the activities of the World Health Organization [27]. As none of these reviews addressed the entire, complex landscape, we undertook a systematic review of all reported health research priority setting initiatives involving LMICs, with a particular focus on methodologies.

## Methods

For peer reviewed articles, the electronic databases PubMed, EMBASE and CINHAL were searched (time period March 2014 or earlier). Reference lists of included articles and review articles were also examined for relevant reports. The following search term combinations were used:

- “priority setting” [all fields] OR “research priorities [All fields] OR “research priority” [all fields] OR “priority research” [all fields] OR “research agenda” [all fields] OR “resource allocation” [all fields] OR “priorities’ [all fields]
  AND
- “global” [key word, MESH] OR “developing country/ies” [keyword, MESH], OR ‘low income countr*” [keyword) OR “middle income countr*” [keyword], OR the name of the 2012 World Bank listed low- and middle-income countries and regions [keyword, MESH] [28]

An initial review of titles and abstracts was undertaken. Articles with a title/abstract that made no mention of health research priority setting, or an activity or outcome that could be described as such were excluded. If insufficient information was provided in the abstract/title to make a determination, the full text was reviewed. Reports were determined to relate to LMICs either if the work was conducted in a low- or middle-income country or region, as defined by the World Bank [28] or the report specified research priorities for LMICs or regions. Articles that reported on priority setting within an organisation were excluded as were reports that described research priorities but provided insufficient detail to determine what process had been used.

A Google search was conducted for non-peer reviewed literature, using the search string ‘health research priority setting’ with the name of each of the World Bank listed LMICs and regions. A search was also undertaken of key websites, including those of the Council on Health Research and Development, Health Research Web, ERA Watch, and the Alliance for Health Policy and Systems Research.

Reports were analysed according to a quality assessment framework, modified from criteria used by the World Health Organization in an earlier review [27], and taking account of the principles of health research priority setting described by Viergever et al [7]. Criteria included who instigated the initiative; what strategy was used; what stakeholders were involved in the process; the outcome of the process; and any evidence of an implementation or follow-up strategy. In initiatives with prioritized outcomes and using classifications used elsewhere [13], [29], [30] the top ten research priorities, for initiatives covering areas other than health systems research, were categorized as either:

- Description (epidemiology or evaluation of existing interventions)
- Discovery (new interventions)
- Development (improving existing interventions)
- Delivery (health policy systems, including cost-effectiveness)

## Results

**Figure 1:**
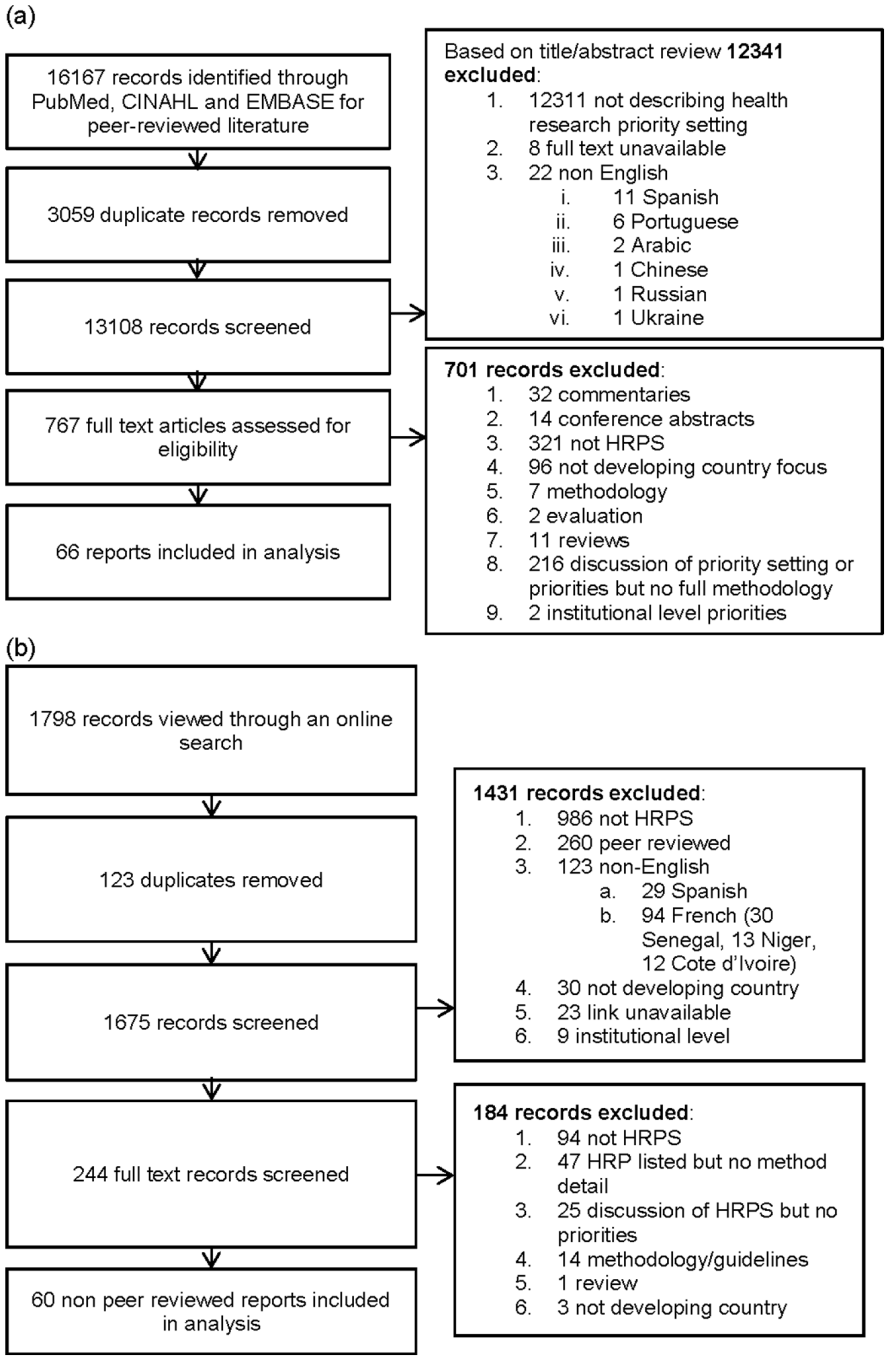
Identification of reports of health research priority setting initiatives from (a) peer reviewed and (b) non peer reviewed sources.

**Figure 2:**
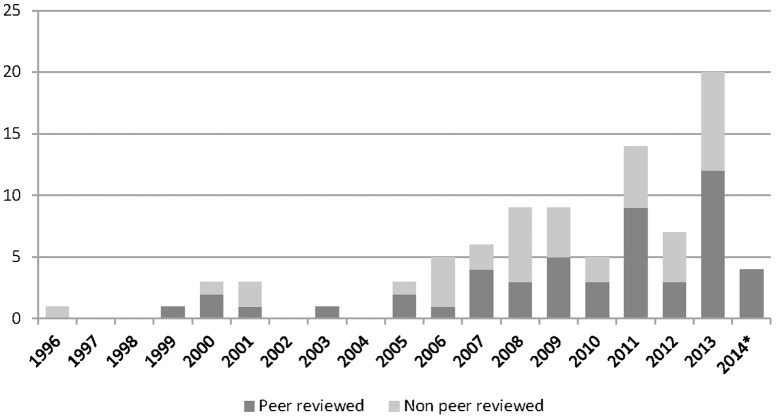
Number of HRPS initiatives per year, peer review and non-peer reviewed.

There were 126 reports on priority setting initiatives that met inclusion criteria (Figure 1, see Table S1 for report details). There were 13 initiatives that were reported on multiples times (together the subject of 48 reports), resulting in a total of 91 separate health research priority setting activities. The number of initiatives per year increased over time, with the highest number in 2013 (Figure 2). As shown in Table 1, initiatives most often described activities at either the global level (46%) or the national level (43%), with a smaller proportion (11%) at the regional level.

**Table 1:** Characteristics of reviewed health research priority setting initiatives with a focus on LMICs.

Characteristic	Category	N (%)	
Setting	Global – LMIC focus	42 (46%)	
	National	39 (43%)	
	Regional	10 (11%)	
Region* (excludes Global)	Sub Saharan Africa	24 (49%)	
	East Asia and Pacific	10 (20%)	
	Latin America and the Caribbean	9 (18%)	
	Middle East and North Africa	6 (12%)	
	South Asia	3 (6%)	
	Europe and Central Asia	1 (2%)	
Income classification* (excludes Global and Regional)	Low income	10 (23%)	
	Lower middle income	17 (44%)	
	Upper middle income	13 (33%)	
Area of health research	All	23 (25%)	
	Communicable diseases	18 (20%)	
	Health systems	11 (12%)	
	Child health	9 (10%)	
	Maternal and reproductive health	8 (9%)	
	Mental health	6 (7%)	
	Non communicable diseases	6 (7%)	
	Other	10 (11%)	
Initiated by*	International organisation or collaboration	42 (46%)	
	LMIC government	29 (32%)	
	Academics – LMIC	14 (15%)	
	Academics – HIC	7 (8%)	
	LMIC government	3 (3%)	
	Consultancy	1 (1%)	
Strategy used*	Conference/workshop	22 (24%)	
	CHNRI	16 (18%)	
	Stepwise	16 (18%)	
	Delphi	11 (12%)	
	ENHR	9 (10%)	
	Survey	7 (8%)	
	CAM	3 (3%)	
	Nominal group technique	3 (3%)	
	COHRED	2 (2%)	
	Concept mapping	1 (1%)	
	Multi-criteria decision analysis	1 (1%)	
	Listening approach	1 (1%)	
Stakeholders involved* – proportion of initiatives engaging relevant stakeholder group	Researchers	100 (100%)	
	Government	70 (74%)	
	Practitioners	50 (55%)	
	NGOs	46 (51%)	
	International organisations	45 (49%)	
	Patients/community	26 (29%)	
	Donors	15 (16%)	
	Private sector	9 (10%)	
Identifies	Broad research areas	32 (35%)	
	Research topics	38 (42%)	
	Specific research questions	21 (23%)	
Research topics	Prioritised	49 (54%)	
	Listed	42 (46%)	
Type of research prioritised		Median	IQR
	Description	25%	10–50%
	Discovery	0%	0–14%
	Development	17%	5–30%
	Delivery	35%	16–64%
Criteria used	Yes	61 (67%)	
	No	30 (33%)	
Decision making	Metric	42 (46%)	
	Consensus	35 (38%)	
	Combination	14 (15%)	
How initial list developed*	Participant nominated	68 (75%)	
	Literature review	26 (29%)	
	Workshop generated	22 (24%)	
	Previous priorities	10 (11%)	
	Other	3 (3%)	
Evidence of implementation/follow-up	Yes	20 (22%)	
	No	71 (78%)	

*Denotes category adds to more than 100% due to classification in a number of ways.

*Region:* 3 initiatives were carried out in multiple regions; *Income classification:* 1 initiative was undertaken in three countries, with different income classifications.

For initiatives at the regional or national setting the largest proportion was from Sub Saharan Africa (49%), followed by East Asia and the Pacific (20%) and Latin America and the Caribbean (18%). Of the 39 national level priority setting initiatives 44% were in lower middle income countries, 33% in upper middle income countries and 23% in low income countries. Research priorities were assessed across all areas of health in 25% of initiatives, communicable diseases in 20%, health systems in 12%, and child health in 10% (Table 1). The number of research priorities identified ranged from 5 to 588, with a median of 29 (IQR 12-55).

The majority of initiatives were instigated by an international organisation or collaboration (46%), by a LMIC government (32%) or LMIC academics (15%). The most common process to elicit priorities was a workshop/conference without any explicit specification of established HRPS methods (24%), followed by CHNRI (18%) and a stepwise process including a literature review, in-depth interviews and consultation (18%). Initial discussions were informed by burden of disease data or literature reviews of existing research in a third of initiatives. All initiatives engaged researchers in the process, with 74% engaging government (including policy makers) and 55% practitioners. The opinions of patients and/or community were formally considered in 29% of initiatives. Research was prioritized (as against just listed) in over half of the initiatives (54%). Of the priority setting initiatives reviewed 42% resulted in specific research topics, 35% in broad research areas, and 23% in specific questions. A small number (8%) provided broad research themes, as well as more specific topics, and in some cases example research questions. The majority of initiatives reviewed (78%) did not provide any evidence of an implementation or follow-up strategy. Of the initiatives covering areas of health other than health systems, 24 provided sufficient information to enable a classification of each research priority. Of the remaining 67 initiatives, 11 covered health systems research, 41 listed research without any indication of priority, and 15 provided research topics with insufficient detail to categorise. Among the top ten priorities in each initiative the median proportion of descriptive research per initiative was 25% (IQR 10–50), discovery 0% (IQR 0–14), development 17% (IQR 5–30) and delivery 35% (IQR 16–64) (Table 1).

The application of criteria to determine research priorities was used in 67% of reports. While not mutually exclusive, the different types of criteria fit into three broad categories (Table 2), criteria at the population level (including burden of disease, equity and efficacy and effectiveness), health systems level (workforce, political context and delivery), and research process and feasibility (knowledge generation, ethics, relevance, funding). Table 2 provides examples of the different criteria used in the initiatives reviewed.

**Table 2:** Type of criteria used for determining health research priorities.

*Type of criteria*	*Examples*
**Population level**	
Burden of disease	• Maximum potential for disease burden reduction [41]
	• Magnitude of the problem [42]
	• Severity of the outcome [43]
	• Size of population benefitting from research [44]
Equity	• Likely equity in achieved disease burden reduction [41]
	• Effect on equity [45]
	• Disparity reduction [46]
Efficacy and effectiveness	• Efficacy and effectiveness [47]
	• Potential of review to influence healthcare practice or policy [48]
**Health systems level**	
Workforce	• Contribution to research capacity strengthening [49]
	• Human resources [50]
Political context	• Government policies [51]
	• Policy relevance [52], [53]
	• Political acceptability [53]
	• Existing international cooperation in a field [54]
Delivery	• Affordable and deliverable [41], [55]
	• Likelihood that intervention affordable to households and governments [48]
	• Cost-effectiveness [56]
	• Effect on efficiency of health system [57]
**Research process and feasibility**	
Knowledge generation	• Avoidance of duplication [58]
	• Innovation [59]
	• Lack of research [60]
Ethics	• Answerability and ethics of research project [41]
	• New knowledge in an ethical manner [33]
Relevance	• Likelihood of review to be relevant to other countries [48]
	• Relevance to economic and social development of the country [54]
	• Importance to developing countries [58]
Funding	• Availability of funds [61]
	• Resources [62]

A number of common challenges emerged in the initiatives including stakeholder engagement, data limitations through limited published information available through literature reviews, and limited capacity to implement research priorities.

## Discussion

This review provides a comprehensive global assessment of published health research priority setting initiatives in LMICs. The majority of priority-setting exercises were conducted at the global level with a LAMIC focus, initiated by international organisations, employed a workshop methodology and focused on prioritised, and specified research topics determined using criteria in the areas of description and delivery across all health research areas. Most did not have any evidence of implementation or follow-up. Earlier reviews have been limited to specific areas of research [19] or geographical areas [17], considered only national initiatives [25], or were restricted to initiatives led by WHO [27]. We found that while the number of health research priority setting initiatives has been increasing over time; there is inconsistent application of methods and outcomes generated and limited evidence, at least in terms of published reporting, of implementation strategies or outcomes. A number of challenges impede research priority setting in LMICs, including appropriate stakeholder engagement as well as data and capacity constraints.

Our review only included initiatives that were reported in English, excluding a number from non-English speaking LMICs. We were also limited by the information provided in the reviewed documents. In some reports only brief information was provided on the strategy used and stakeholders engaged, suggesting a possible lack of transparency of the process. Some reports did note that participant confidentiality was essential to ensure unbiased opinions were provided. Beyond the reports reviewed here, we found indirect or secondary evidence of other priority setting activities in LMICs for which we were unable to locate a publicly available report, or links to which were disabled. It is also likely that we missed information on other activities with no published information available that were therefore not searchable.

The increase over time in the number of initiatives may be due to a greater focus on health research more generally in LMIC settings. While the 10/90 gap may have closed somewhat since 2000, the concept continues to provide motivation for increased health research in LMICs. The 2005 Paris Declaration [31] to guide more effective aid and development programs has also encouraged alignment with national priorities and processes. It is increasingly acknowledged that health research priority setting facilitates targeted research that has the potential for the greatest impact [7] as well as building national capacity in a number of respects. With finite resources and increasing demands on health systems due to the double burden of disease in LMICs [32], it is understandable that there is a heightened sense of urgency about identifying health research priorities.

One of the critical aspects of priority setting is achieving the right level of detail in the research priorities, too broad and they fail to provide guidance, too detailed and they risk being too prescriptive. Global level exercises pose the additional challenge of the application of priorities to a variety of contexts, as noted by Kosek [33]. Some of the initiatives reviewed provided broad research themes, with sub-themes providing more detail, and examples of specific questions, which may facilitate implementation. Whether research options are prioritized or just listed is another important feature of research priority setting. Our review demonstrated that over half of the outcomes were prioritized, with the majority resulting in research topics or specific research questions. Lack of prioritization risks preferential selection of research that is easier to implement, or more closely aligned with current activities, rather than the research that is most urgent.

It has been asserted that existing interventions have the potential to provide many of the tools required to address poor health outcomes in LMICs [34], and the knowledge gap is in implementation research rather than in discovery of new technologies. Our review provides evidence that this perspective is being recognized in priorisation processes, with a median of 0% per initiative falling into the category of discovering new interventions and 35% related to delivery research.

The earlier analysis by Rudan et al highlighted the importance of defined criteria for priority setting, stakeholder input, and the translation of research into policy as well as emphasising the need for greatly strengthening capacity to drive and implement research in LMICs [19]. It is therefore encouraging that of the initiatives covered in our review the majority used criteria to determine research priorities (67%). While recognising the limitations of the available tools for health research priority setting, Rudan et al also emphasized the importance of their use [19]. Our review indicates an inconsistent application of available established methods of HRPS, potentially hindering repeatability and transparency of the process. The review of selected national health research priority setting initiatives by Tomlinson et al highlighted similar concerns regarding limited evidence of implementation and engagement with stakeholders, as well as how audiences were targeted [25]. Likewise our review demonstrates a high level of engagement with researchers and government, but less involvement of other key stakeholders. The WHO review noted that the use of any established strategy was rare, with similar results reflected here, with less than half of the initiatives using an established strategy. While it is unlikely that there will ever be a ‘gold standard’ method for health research priority setting, the application of one of the recognised strategies provides a framework for carrying out the process that ensures inclusiveness, defined criteria for determining priorities, and transparency of process [7], [27].

There are challenges in the process of research priority setting regardless of the approach used, demonstrated through this review. A number of the initiatives noted that initial literature reviews to determine burden of disease and current research activities, were of limited use due to the lack of country specific information. Regional and global estimates are often used as an alternative, but do not take into account the unique situations of individual countries [35]. There is a large body of evidence that demonstrates that data gaps are an inherent part of health systems in LMICs and highlight the need for country level research on burden of disease [8], [36]. The priority given to epidemiological research in the initiatives analysed also highlights a need to better understand the burden of disease in LMICs. Limited health research capacity is an ongoing issue for LMICs and reduces the ability to implement research priorities. Capacity constraints have been reinforced by a legacy of research being undertaken by high income country academics [36], and brain-drain eroding national research capacity [37]. Multi-bi financing may also be further contributing to reduced research capacity, as activities are narrowed and may target short-term outcomes rather than long-term public health sustainability [3]. Focused attention on capacity constraints and opportunities for capacity building at both the individual and institutional level, will contribute to improved implementation of priority research and overall health improvements [36], [38].

Involvement of a wide range of stakeholders in the health research priority setting process has been identified as a way of both ensuring legitimacy and inclusiveness of the approach [7], [39] and of driving health equity [7]. Poor stakeholder engagement may lead to opinion bias, noted in a number of initiatives reviewed, relating to health experts consciously or unconsciously preferencing research fields that are familiar to them [4]. Kapriri has documented the difficulties in engaging with a wide range of stakeholders but also stresses how important it is [39]. Challenges with engagement also link to research capacity, with required engagement of technical experts often limiting involvement to experienced developed country researchers, potentially resulting in bias away from national health priorities [4]. While researchers and government were well represented in the initiatives reviewed, affected populations (patients and civil society) had far less involvement. The increased participation of donors (only 16% in reviewed initiatives) may also strengthen links between prioritised research and funding opportunities.

An implementation strategy is essential for ensuring that the outcomes of health research priority setting exercises are translated into research projects that ultimately improve the health of LMIC populations. Without evidence of implementation or health outcomes it is difficult to assess the quality of research priority setting exercises [40]. While the initiatives reviewed drew attention to the issue of implementation and follow-up, expressing optimism that their analysis would inform research agendas, few reports described concrete strategies for achieving this goal, let alone attempted to measure whether it had been achieved. This indicates a need for improved dialogue among instigators of research priority setting, governments, research institutions, and funding bodies. Improved documenting of priority setting would also enhance assessment of health outcomes, while enabling LMICs to draw on the experiences of others. What is still missing is critical review of the output and implementation of health research priorities, and the way in which they address the criteria under which they were set.

## Conclusion

While a focus on global health initiatives, such as those to eradicate specific diseases, has provided momentum and financial support, it should not be at the expense of national health priorities [3], [4]. Health research priority setting in LMICs is aimed at directing limited resources to areas of greatest need and impact. While workshops with no explicit application of established health research priority setting methods was the most common approach, the use of established strategies to determine priorities currently provide the most useful tools to ensure conduct in a transparent and repeatable manner. Despite most initiatives highlighting the importance of dissemination and implementation of priorities, there was limited evidence of strategies to do so. Without evidence of implementation and ultimately health outcomes, it remains challenging to assess the quality and impact of health research priority setting strategies in LMICs.

## Supporting Information

Table S1 Details of health research initiatives.
(XLSX)
